# The cognitive and behavioral correlates of functional status in patients with frontotemporal dementia: A pilot study

**DOI:** 10.3389/fnhum.2023.1087765

**Published:** 2023-02-22

**Authors:** Electra Chatzidimitriou, Panagiotis Ioannidis, Despina Moraitou, Eleni Konstantinopoulou, Eleni Aretouli

**Affiliations:** ^1^Laboratory of Cognitive Neuroscience, School of Psychology, Faculty of Philosophy, Aristotle University of Thessaloniki, Thessaloniki, Greece; ^2^B Department of Neurology, AHEPA University Hospital, Aristotle University of Thessaloniki, Thessaloniki, Greece; ^3^School of Psychology, University of Ioannina, Ioannina, Greece

**Keywords:** cognition, neuropsychological performances, behavior, frontotemporal dementia, functional status

## Abstract

**Objective**: Frontotemporal dementia (FTD) impinges significantly on cognition, behavior, and everyday functioning. Goal of the present study is the detailed description of behavioral disturbances and functional limitations, as well as the investigation of associations between cognition, behavior, and functional impairment among FTD patients. Given the importance of maintaining a satisfying functional status as long as possible, this study also aims to identify the cognitive correlates of compensatory strategy use in this clinical group.

**Methods**: A total of 13 patients diagnosed with FTD (behavioral variant FTD = 9, non-fluent variant primary progressive aphasia = 3, semantic dementia = 1) were administrated a broad range of neuropsychological tests for the assessment of different cognitive abilities. Behavioral symptomatology and performance on everyday activities were rated with informant-based measures. Descriptive statistics were used for the delineation of behavioral and functional patterns, whereas stepwise multiple regression analyses were performed to identify associations between cognition, behavior, and functional status.

**Results**: Negative symptoms, especially apathy, were found to predominate in the behavior of FTD patients. Instrumental tasks, such as housework and leisure activities, appeared to be the most impaired functional domains. Working memory was the strongest cognitive correlate of performance across various domains of everyday functioning, whereas working memory along with short-term verbal memory accounted for a great proportion of variance in compensatory strategy use. Behavioral disturbances and especially negative symptoms were also found to contribute significantly to functional impairment in FTD.

**Conclusions**: Executive dysfunction, as well as behavioral disturbances contribute significantly to functional disability in FTD. Early interventions tailored at these domains may have the potential to improve functional outcomes and delay the rate of functional decline among FTD patients.

## Introduction

Frontotemporal dementia (FTD) is a common cause of young-onset dementia in individuals under 65 years of age. It constitutes an umbrella clinical term for a heterogeneous group of neurodegenerative diseases characterized by progressive decline in social behavior, personality, executive function and/or language (Bang et al., 2015; Moheb et al., 2017). FTD is classified into three clinical variants/subtypes: (1) behavioral-variant FTD (bvFTD); (2) semantic-variant primary progressive aphasia (svPPA) or semantic dementia (SemD); and (3) non-fluent variant primary progressive aphasia (nfvPPA; Bang et al., 2015). Various underlying neuropathological mechanisms can lead to the FTD clinical phenotype, all of which are characterized by the selective degeneration of the frontal and temporal cortices (Bang et al., 2015).

FTD has a devastating effect on activities of daily living (ADLs; Mioshi et al., 2007; Wicklund et al., 2007; Mioshi and Hodges, 2009; O’Connor et al., 2016a,b). Approximately 5 years after symptom onset, the vast majority of bvFTD patients tend to develop severe functional impairment (Mioshi et al., 2007). However, information on the functional status of patients with FTD is relatively sparse and focuses mainly on the behavioral-variant of FTD (Josephs et al., 2011; Lima-Silva et al., 2013, 2015a,b; Musa Salech et al., 2022). Research has shown that, as happens in AD, FTD initially affects instrumental activities of daily living (IADLs), while decline in basic activities of daily living (BADLs) develops later in the course of the disease (Mioshi et al., 2010; Musa Salech et al., 2022). In comparison to other types of dementia, such as AD, bvFTD has been associated with more profound functional impairments (Roberson et al., 2005; Borroni and Padovani, 2007; Mioshi et al., 2007). In a recent study that compared the trajectories of everyday functioning in patients with FTD and AD, findings showed that patients with bvFTD were significantly more impaired on everyday tasks, such as finance management and engaging in leisure activities, although they experienced milder cognitive deficits (Giebel et al., 2021).

The rate of functional decline can vary significantly among FTD individuals, making prognosis very difficult (Josephs et al., 2011). Therefore, it is essential that cognitive and behavioral correlates of functional status in these patients be known. Cognitive correlates of ADLs in FTD are relatively unknown and knowledge in this research area is derived mainly from studies that examine predictors of functional status in other clinical groups, especially in patients with AD or in groups at high risk for developing dementia (e.g., MCI; Farias et al., 2003; Aretouli and Brandt, 2010; Schmitter-Edgecombe and Parsey, 2014; Moheb et al., 2017).

Executive dysfunction and decline in general measures of cognitive functioning (e.g., Mini-Mental State Examination) have been associated with a lower ability to undertake IADLs in older adults with varying degrees of cognitive impairment (Pereira et al., 2008). Furthermore, executive functions (Bell-McGinty et al., 2002; Cahn-Weiner et al., 2002) and memory skills (Goldstein et al., 1992) have been correlated with ADL performance, in both cognitively intact and impaired geriatric individuals. These findings suggest that both executive functions and memory contribute substantially to functional capacity. In the matter of specific components of executive cognition that relate to functional status, studies have yielded mixed results. Some authors have argued that inhibitory control, mental flexibility, psycho-motor speed, and sequencing ability contribute significantly to IADLs (Bell-McGinty et al., 2002; Cahn-Weiner et al., 2002; Jefferson et al., 2006), whereas others have suggested that measures of planning and problem solving are stronger correlates (Lewis and Miller, 2007).

As mentioned above, the literature examining cognitive correlates of functional status specifically in FTD patients is, to date, very limited (Josephs et al., 2011; Moheb et al., 2017; Musa Salech et al., 2022). In all three FTD subtypes, decreased performance on measures of executive functions is correlated with poorer IADL performance. Furthermore, memory deficits were found to contribute significantly to IADLs, but only in the bvFTD and nfvPPA subgroups (Moheb et al., 2017). It is also noteworthy that difficulties in IADL tasks were not correlated with performance on measures of language functions in none of the three FTD subgroups, absurdly neither in the language variants of the disease (Moheb et al., 2017).

In addition to cognitive deficits, behavioral disturbances have also been reported to predict functional status in FTD, as more severe behavioral symptoms are correlated with increased functional disability (Moheb et al., 2017; Musa Salech et al., 2022). More specifically, apathy was found to be the most robust behavioral correlate of functional impairment in both bvFTD and SemD (Kipps et al., 2009; O’Connor et al., 2016a; Musa Salech et al., 2022). Stereotypical behavior and disinhibition has also been associated with poor functional outcomes, but only in bvFTD (O’Connor et al., 2016b; Musa Salech et al., 2022). With respect to other behavioral symptoms that correlate with the functional status in FTD, literature suggests that hallucinations and anxiety are associated with decreased functional capacity in bvFTD, whereas delusions and eating disorders seem to contribute to functional decline in nfvPPA (Moheb et al., 2017). When it comes to depressive symptoms, evidence suggests that, in bvFTD, the severity of depressive symptoms is associated with the severity of functional deficits in a negative way (Moheb et al., 2017). In other words, as bvFTD progresses, depressive symptoms tend to decrease or remain stable, rather than increase, possibly due to loss of insight (Diehl-Schmid et al., 2006; Ranasinghe et al., 2016).

In conclusion, FTD impinges significantly on cognition, behavior, and everyday functioning. Notwithstanding, studies that focus on the profile of behavioral disturbances and functional deficits, as well as the cognitive and behavioral correlates of functional disability among these patients are to date very limited, indicating a research gap (Rascovsky et al., 2005; Mioshi et al., 2007; Mioshi and Hodges, 2009; Mioshi et al., 2010; Josephs et al., 2011; Lima-Silva et al., 2013; O’Connor et al., 2016a,b; Moheb et al., 2017; Giebel et al., 2021; Musa Salech et al., 2022).

The first objective of the present study was to investigate the profile of functional deficits and behavioral disturbances among FTD patients. Regardless of the subtype, FTD has a devastating effect on behavior and activities of daily living. However, the exact behavioral and functional changes are not yet clearly documented. Delineating the behavioral disturbances and daily functioning restrictions in FTD is of great clinical importance for both diagnostic and therapeutic purposes.

The second objective was to identify the cognitive and behavioral correlates of everyday functioning, given that cognitive deficits and behavioral changes constitute major factors that contribute to the functional status in dementia syndromes (Giebel et al., 2021). Previous studies have focused mainly on patients with MCI or AD and especially on the predictive role of executive functions for everyday functioning. Only a few similar studies in FTD exist, despite the fact that these patients demonstrate prominent behavioral symptoms and significant functional impairment. The present study aimed to investigate not only executive functions, but instead a wide spectrum of neuropsychological performances (visuospatial abilities, visual and verbal short-term and long-term memory skills, language abilities, and executive functions), as well as behavioral symptoms in relation to functional disability in individuals with FTD. Moreover, an additional objective was to specify the cognitive correlates of behavioral disturbances in this clinical group, taking into consideration that cognitive deficits may play a decisive role on the presentation of behavioral disorders.

A final goal of the present study was to identify cognitive factors that could reflect the use of compensatory strategies in everyday tasks in patients with FTD. This particular parameter has not been investigated before, as far as we know. Many scales have been developed to capture functional difficulties in IADLs in individuals with dementia (Reisberg et al., 2001; Farias et al., 2008). However, only a few of them take into consideration the use of compensatory strategies (Schmitter-Edgecombe et al., 2014). Compensatory strategies (e.g., to-do lists, memory notebook, alarms, GPS, etc.) can assist individuals and increase their functional independence (Schmitter-Edgecombe et al., 2014). Maintaining a satisfying functional status as long as possible is undoubtedly of great importance (Tomaszewski Farias et al., 2018). Understanding how and under what circumstances individuals use compensatory strategies to overcome or mitigate cognitive challenges is therefore critical (Schmitter-Edgecombe et al., 2014), since such knowledge could guide treatment planning (for example, providing guidelines for a cognitive rehabilitation program that focuses on the use of compensatory strategies; Schmitter-Edgecombe et al., 2014).

Based on previous findings and the neuropathology of the disease, we hypothesized: (a) that performance on executive function and memory measures will be the strongest cognitive correlates of functional status among FTD patients, compared to other measures of cognitive functioning. We also assumed; (b) that more severe behavioral disturbances will be associated with more pronounced functional impairment. Furthermore, we expected; (c) that negative symptoms, such as apathy, will contribute significantly to functional outcomes. Finally, with respect to our last research goal, we assumed; and (d) that performance on both executive cognition and memory measures will be correlated with compensatory strategy use among FTD patients.

## Methods

### Participants

A total of 13 individuals diagnosed with FTD (bvFTD = 9; nfvPPA = 3; svPPA = 1) and their caregivers were recruited from the 2nd Neurology Clinic of AHEPA University General Hospital of Thessaloniki between October 2019 and July 2020. Study participants were informed of the study and assessed during their scheduled visit at the outpatient clinic. Diagnoses of FTD were made by an expert neurologist and a neuropsychologist. In addition, neuroimaging data (such as, MRI and SPECT) were collected from all the patients as part of the diagnostic process.

In the matter of sociodemographic information, almost two thirds of the patients were males (*N* = 8; 61.5%). Their mean age was 67 years (±9.13) and their average years of education were 12.46 (±4.67). The mean disease duration was 3.38 years (±1.71). Three patients (23%) had a positive family history of dementia. Demographic characteristics of the participants are summarized in Supplementary Table 1.

Patients were included in the study if they: (1) met criteria for FTD (Neary et al., 1998); (2) had a close relative or friend informant who could reliably report on their behavior and everyday functioning; (3) did not have a physical disability that could influence ability to undertake activities of daily living; and (4) did not have major depression. Duration of disease was not an exclusion criterion and was estimated based on the onset of symptoms, as reported by the informants during the interview session.

Neuropsychological assessments of patients were performed on approximately two 2-h sessions. These sessions included the administration of a broad range of neuropsychological tests evaluating visuospatial abilities, memory skills, language abilities, and executive functions. In addition to traditional paper-based tests, a computerized battery assessing different aspects of executive functioning was also administered. It is noteworthy that some of the neuropsychological measures described in the study were used as part of the diagnostic procedure.

Caregivers were interviewed in the clinic usually in one session lasting approximately 2 h. Most of them were spouses of the patients (77%) and 23% were adult children. During the session, three informant-based scales assessing patient’s behavior and everyday functioning were administered through clinical interview.

### Protocol approvals and patient consents

This study was reviewed and approved by the Bioethics and Ethics Committee of Medical School of Aristotle University of Thessaloniki, Greece. Written informed consents was obtained from all the participants and primary caregivers before entering the study.

### Procedures

#### Cognitive assessment

A wide range of neuropsychological tests were administered to the participants for the assessment of different cognitive domains, such as executive functions, episodic short-term and long-term verbal and visual memory, visuospatial abilities, and language. However, given that deficits in executive functions predominate in the clinical presentation of FTD patients, greater emphasis was placed upon the thorough assessment of executive cognition. Below, there is a brief description of the neuropsychological tests administered. A summary of the neuropsychological tests that were used for the assessment of each cognitive domain is included in the Supplementary Material (see Supplementary Table 2).

### 1) Executive functions

Executive functions were assessed with both traditional (paper-based) and computerized neuropsychological tests. The following domains of executive cognition were evaluated: working memory/updating, inhibition, set-shifting, and verbal fluency. With respect to paper-based tests, some of the most widely used executive tests, as well as a brief executive function battery were administered to participants. In addition, computerized neuropsychological tests from the “NIH-EXAMINER” battery (Executive abilities: Measures and Instruments for Neurobehavioral Evaluation and Research, Kramer et al., 2014) were also included.

### Working memory—updating

#### Digit span test

The Digit Span test (Wechsler, 1997) was administered for the assessment of verbal short-memory and working memory. The version of the test that was used in the present study consists of two parts (forward and backward digit span) and is part of the “Neuropsychological Battery” of the Laboratory of Cognitive Neuroscience of Aristotle University of Thessaloniki (AUTH), Greece (Kosmidis et al., 2011). In both parts of the test, higher scores indicate better performance.

#### Dot counting test

The Dot Counting test (Kramer et al., 2014) constitutes a subtest of the computerized neuropsychological battery NIH-EXAMINER and measures the updating information ability in working memory and, specifically, verbal working memory. Credit is given based on how many totals the examinee can recall correctly from each trial. The total score is produced by summing the correctly recalled items over the six experimental trials (range: 0–27).

#### N-back test

The n-back test (Kramer et al., 2014) is a commonly used test for the assessment of working memory, that requires flexible updating capabilities. The n-back test that was used in the present study is included in the NIH-EXAMINER battery and consists of a 1-back and a 2-back spatial tasks to assess spatial working memory. However, only the 1-back task was administered to patients, because the 2-back task is too challenging for the specific clinical group. The 1-back task consists of one block of 30 trials. The total number of correct trials, as well as the mean response time of correct trials were included in the analyses of the present study.

### Inhibition

#### Flanker test

The Flanker Test (Kramer et al., 2014) is a commonly used test that measures inhibition of prepotent responses. The Flanker test administrated in the present study is included in the NIH-EXAMINER battery. The test consists of a total of 48 trials. During the test, the examinee is presented with two different conditions, congruent and incongruent. The following variables were included in the analyses of the present study: (1) the total number and the mean response time of correct trials for both conditions; (2) the total number and the mean response time of correct congruent trials, as well as; and (3) the total number and the mean response time of correct incongruent trials.

#### Continuous performance test (CPT)

The Continuous Performance Test (Kramer et al., 2014) is a classic computerized response inhibition task which requires subjects to respond to a certain type of stimulus and withhold a response to another. The CPT test administrated in the present study is included in the NIH-EXAMINER battery. The task consists of 100 trials, 80% of which demonstrate the target image. The non-target images that are presented, are of a similar shape and of comparable size to the target. For the purposes of the present study, the following variables were included in the analyses: (1) the total number of trials for both target and non-target conditions where the subject’s response (or non-response) was correct; (2) the total number and the mean response time of correct target condition trials; (3) the total number of non-target condition trials where the subject—correctly—did not respond and, finally; and (4) the total number of trials where the subject—wrongly—provided more than one response.

#### Stroop test

The Stroop Test (Stroop, 1935) included in the present study consists of three parts. The first two parts of the test assess visual attention and processing speed. The third part evaluates the ability to inhibit prepotent (automatic) in favor of more appropriate responses and, more specifically, assesses the ability to suppress word reading in favor of color naming (inhibitory control; Stroop, 1935). The total number of words uttered in each condition constitute the three sub-scores obtained from this test. Higher scores indicate better performance. Finally, based on the performance on the three tasks, an interference index was computed to show the extent to which performance on the color-word condition was affected by interference.

### Set-shifting

#### Set-shifting test

The Set-Shifting Test (Kramer et al., 2014) measures the ability to switch between mental sets by altering behavior and actions due to changing conditions. It is also a task related to cognitive flexibility. The Set-Shifting Test that was used in the present study is included in the NIH-EXAMINER battery. The task is organized into three blocks: homogenous block A, homogenous block B, and heterogeneous block AB (the shift block). For the purposes of the present study, the following variables were included in the analyses: (1) the total number and the mean response time of correct trials in the homogenous blocks A and B; (2) the total number and the mean response time of correct trials in the shift block AB; (3) the total number and the mean response time of correct shifted trials in the shift block; (4) the total number and the mean response time of correct non-shifted trials in the shift block, as well as; and (5) the switching cost (estimated by subtracting the mean response time in the homogenous blocks A and B from the mean response time in the shift block), a variable that indicates to what extent performance is disrupted in tasks that involve set-shifting.

#### Trail making test (TMT)

In the present study, the Greek version of the Trail Making Test was administered to participants (Reitan, 1958; Kosmidis et al., 2011). Part A was used for the evaluation of visual attention and visuo-motor speed, whereas Part B was used for the assessment of set-shifting and cognitive flexibility. Scoring in both parts is expressed in terms of the total time required to complete the task. The less time one needs, the better his performance. It has also been suggested that a ratio score of part B to part A may have significant utility as measure of frontal executive function (Martin et al., 2003). For that reason, except for the completion time of the tasks, ratio scores were also included in the analyses.

### Verbal fluency

#### Verbal fluency test

Tests of verbal fluency evaluate an individual’s ability to retrieve specific information within restricted search parameters. For the assessment of verbal fluency, the Greek version of the verbal fluency test was administered to participants (Kosmidis et al., 2011). This test consists of two parts: the semantic/category and the phonemic verbal fluency task. In both parts of the test, higher scores indicate better performance. Two sub-scores, representing the total number of correct words produced in each task of the test, were included in the analyses of the present study.

In addition to the aforementioned neuropsychological tools, a brief comprehensive battery of executive functions was administered.

#### FRONTIER executive screen (FES)

The FRONTIER Executive Screen (FES) (Leslie et al., 2016) is a brief executive screening tool (duration: 10–15 min) that was developed primarily in order to differentiate FTD from AD (Leslie et al., 2016). In the present study, the Greek adaptation of the test was used. The FES consists of three subtests that assess verbal fluency, verbal inhibitory control, and verbal working memory. Each subtest generates a score from 0 to 5. Total FES score varies from 0 to 15 and is produced by summing the scores for the three subtests. Higher scores indicate better executive abilities.

### 2) Visuospatial abilities and visual memory

#### Taylor complex figure test

The Taylor Complex Figure test (Taylor, 1959) is a constructional memory task and was administered for the evaluation of visuospatial ability, perceptual organization, visual episodic short-term and long-term memory, as well as recognition of visual information. In the present study, four conditions of the test were administered to the participants (copy, immediate recall, delayed recall, and recognition). In all four conditions, higher scores indicate better performance.

### 3) Verbal memory

#### Story memory test

The Story Memory test is included in the “Neuropsychological Battery” of the Laboratory of Cognitive Neuroscience of AUTH (Kosmidis et al., 2011) and was used as a measure of verbal memory. It consists of two conditions (immediate and delayed recall) that assesses episodic short-term and long-term verbal memory of semantically related information. The two sub-scores obtained from this test relate to the performance on the task of immediate recall (sum of the items correctly recalled in the two trials of immediate recall, range: 0–32) and the task of delayed recall (range: 0–16).

#### Word learning test

The Word Learning test is, also, included in the “Neuropsychological Battery” of the Laboratory of Cognitive Neuroscience of AUTH (Kosmidis et al., 2011) and was used as a measure of verbal memory. It consists of three conditions (immediate recall, delayed recall, and recognition) and examines episodic short-term and long-term verbal memory of information that are not semantically related to each other. In the matter of final score, three sub-scores are obtained from the Word Learning Test and they relate to performance on the task of immediate recall (sum of the words correctly recalled in the four trials of immediate recall, range: 0–40), the task of delayed recall (range: 0–10), and, finally, the task of recognition (range: 0–20).

### 4) Language

#### Confrontation naming test

Confrontation naming is a language task that assesses word-finding difficulties or anomia. The version of the test used in the present study is included in the “Neuropsychological Battery” of the Laboratory of Cognitive Neuroscience of AUTH (Kosmidis et al., 2011) and evaluates noun retrieval abilities. It consists of 40 pictures of objects from a variety of semantic categories and the examinee is asked to name the displayed items. The final score in this test results from the sum of the correctly named items (range: 0–40).

### 5) Global cognitive function

#### Clock drawing test (CDT)

The Clock Drawing constitutes a widely used neuropsychological test for the assessment of the mental status of patients with various neurological and psychiatric disorders (Freedman et al., 1994). The CDT version included in the present study is part of the “Neuropsychological Battery” of the Laboratory of Cognitive Neuroscience of AUTH (Kosmidis et al., 2011) and was used as a measure of the global cognitive ability, since the task of drawing a clock entails several cognitive processes (semantic memory, intact attention, receptive language, visuo-spatial abilities, visuo-constructional skills, as well as executive functions, such as planning, organization, and self-monitoring; Bozikas et al., 2008). In CDT, the total score ranges from 0 to 15 and higher scores indicate better performances.

### Behavioral assessment

#### Frontal behavioral inventory (FBI)

The Frontal Behavioral Inventory (FBI) (Kertesz et al., 1997, 2000; 2003) is a 24-item quantifiable questionnaire directed to the caregiver. It was developed and standardized in order to differentiate FTD from other dementias and to quantify the severity of behavioral disturbances in FTD. The FBI was constructed based on the core diagnostic features of FTD, as reflected by the Neary et al. (1998) criteria. It requires a reliable informant for a face-to-face interview and its administration lasts about 20–30 min, depending on the severity and extent of patient’s symptoms. In the present study, the Greek adaptation of the scale was used. The questionnaire consists of 12 items that assess negative behaviors/deficits and 12 items that evaluate positive behaviors, related to disinhibition. Higher total scores represent greater behavioral changes. In the present study, except for the total score and the score for each item separately, sub-scores for the negative and positive symptoms were also included in the analyses.

### Functional assessment

#### Disability assessment for dementia (DAD)

ADLs were assessed with the Disability Assessment for Dementia (DAD) (Gélinas et al., 1999), an informant-based scale that consists of 40 items. DAD includes 17 items related to basic ADLs and 23 items related to instrumental ADLs. Basic ADL items assess hygiene, dressing, continence, and eating, while instrumental ADL items evaluate meal preparation, telephoning, going on an outing, finance and correspondence, medication, leisure activities, and housework. The final total score is reported as a percentage. Scores vary from 0 to 100 and lower DAD scores indicate greater functional impairment. In this study, except for the total DAD score, BADL and IADL sub-scores, as well as sub-scores for each functional domain separately, were included in the analyses for a thorough investigation of functional limitations in FTD patients.

#### Instrumental activities of daily living-compensation scale (IADL-C)

The Instrumental Activities of Daily Living-Compensation scale (IADL-C) (Schmitter-Edgecombe et al., 2014) was developed to capture early functional difficulties and to quantify compensatory strategy use in older individuals with cognitive deficits. The IADL-C was translated and backtranslated into Greek, for the purposes of the present project. The scale constitutes a 27-item instrument that includes questions pertaining to activities of four functional domains: money and self-management, home daily living, travel and event memory, and social skills. The IADL-C is rated according to a 10-point response scale. Based on the research goals of the present study, greater emphasis was placed upon ratings related to compensatory strategy use. A percentage related to the number of activities for which compensatory strategies are deployed by the participant, in relation to the total number of IADL activities of the questionnaire in which the participant is involved, was computed, and included in the analyses of the present study. Finally, it is noteworthy that the IADL-C is composed of two versions, one for the participant/patient and one for his informant. In this study, only the informant-rated questionnaire was included, since FTD patients often demonstrate limited insight into their difficulties and, therefore, they are not considered reliable raters.

#### Data analysis

Statistical analyses were performed using SPSS 25 for Windows. Descriptive statistics were used for an overall description of the behavioral disturbances and functional deficits of FTD patients. More specifically, variables related to the scores obtained from FBI were recoded into new, dummy variables (0, 1) for the estimation of frequencies of none at all/mild (coded with 0) and moderate/severe (coded with 1) behavioral symptoms. Moreover, for the estimation of appearance rate of major negative and positive behavioral symptoms, dummy variables (0, 1) were created with two categories: scores from 0 to 12 (coded with 0) and scores from 13 to 36 (coded with 1). For the estimation of significant total behavioral symptoms, FBI scores were also recoded into new, dummy variables (0, 1) with two categories: scores from 0 to 24 (coded with 0) and scores from 25 to 72 (coded with 1). In addition, means were used to examine participants’ mean performance on specific domains of BADLs and IADLs, as well as on total ADLs.

The contribution of cognitive and behavioral symptomatology to everyday functioning among FTD patients was investigated with a series of stepwise multiple linear regression analyses. The selection of variables that were entered in the regression analyses was based on the correlations that resulted from Spearman’s non-parametric tests, that were used due to the small sample size. In addition, because each of the FTD subgroup was quite small, we did not perform the regression analyses on the three subgroups separately.

In the first set of analyses, the relative contributions of measures of different cognitive domains (e.g., executive functions, verbal and visual memory, visuospatial abilities, and language) to DAD scores were investigated in stepwise models, since our first goal was to examine which neuropsychological tests of each cognitive domain have the greatest unique contribution to functional status. Afterwards, based on the statistically significant predictive factors that resulted from the first set of analyses, we examined which cognitive ability has the greatest unique contribution to each functional domain. We followed a variable reduction approach, entering into our final sets of stepwise regression analyses only one variable from each cognitive domain to predict performance on tasks of daily living (the one, that after competing with the other variables of the same cognitive domain showed the highest correlation with everyday functioning).

Of note, we investigated the cognitive and behavioral correlates of only those functional domains that were reported to demonstrate most prominent deficits. Therefore, we selected to enter in the analyses as dependent variables only the domains of everyday functioning for which the mean performance of participants was under 80% compared to their previous level of functioning, as described by knowledgeable informants. In this way, dressing, eating, and continence were left out from the regression analyses.

Furthermore, we examined the cognitive correlates only in relation to the behavioral disturbances that predominated among FTD patients. Thus, we selected to include in the analyses only moderate-to-severe behavioral symptoms that were reported by at least 60% of knowledgeable informants. In this way, only apathy, aspontaneity, inflexibility, inattention, logopenia, loss of insight, irritability, and obsessions were included, whereas the remaining 16 FBI behavioral symptoms were omitted from the regression analyses.

## Results

### Behavior and everyday functioning in FTD

**Figure 1:**
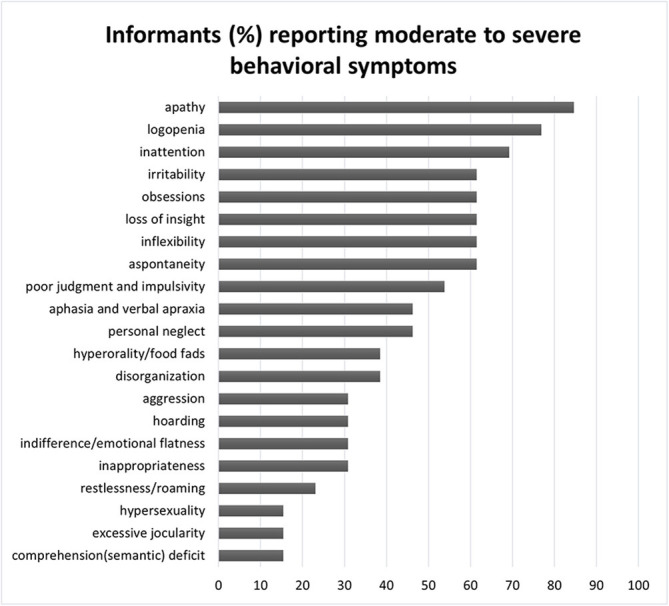
Frequency of moderate-to-severe behavioral symptoms in FTD patients as reported by knowledgeable informants. FTD, frontotemporal dementia.

Descriptive statistics summarized the most commonly reported behavioral symptoms, as well as the domains of everyday functioning that FTD patients encounter the greater difficulties. Figure 1 illustrates the frequency of moderate-to-severe behavioral symptoms among participants based on informants’ reports on the FBI.

Apathy constitutes the most prevalent behavioral symptom displayed in the vast majority of FTD patients (approximately 85%) and it is followed by logopenia and inattention that are reported by approximately 77% and 70% of informants, respectively. Generally, negative symptoms were found to be much more prevalent than positive among FTD participants, since the former were reported more than twice as much as the latter by knowledgeable informants (76.9% compared to 30.8%). In addition, significant overall behavioral symptomatology was reported by more than half of informants (54%).

**Figure 2:**
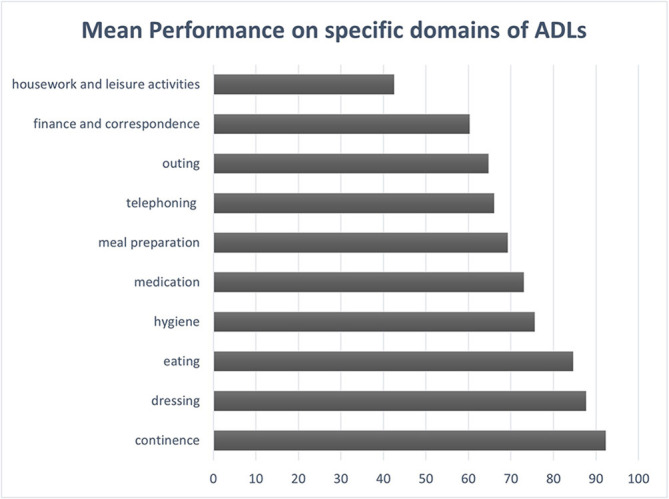
Mean level of performance on specific domains of ADLs in FTD patients, based on reports provided by knowledgeable informants. ADLs, activities of daily living.

With regards to functional status, the mean level of performance on different activities of daily living, as rated by knowledgeable informants, is delineated on Figure 2. It is worth mentioning that higher scores, approaching 100, represent better performance, whereas lower scores indicate functional decline. As shown in the figure, housework and leisure activities constitute the most impaired domains of everyday functioning among patients with FTD, since performance on these areas has been reduced by almost half (43%), compared to the previous level of functioning. Finance and correspondence are the next most affected domains with a mean level of performance at 60%. BADLs such as eating, dressing, and continence appear to be relatively preserved (85%, 88%, and 92%, respectively). FTD patients demonstrate substantial overall functional decline compared to their previous level of everyday functioning, observed mainly on IADLs compared to BADLs, therefore significantly impinging on their independent functioning.

### Cognitive correlates of functional status in FTD

The potential contribution of the neuropsychological tests to functional status was evaluated with stepwise multiple regression analyses. In the first set of analyses, it was found that among all the neuropsychological tests administered, only the working memory subtest of the FES, the total score of the FES, the phonemic verbal fluency test, and the recognition task of the list learning test contributed significantly to ADL-score, whereas the remaining tests did not contribute to ADL performance, as assessed by the DAD ratings. As shown in Table 1, in the second set of stepwise regression analyses, it was found that the working memory subtest of the FES had the greatest contribution to ADLs, accounting for 68.4% of variance in everyday functioning [*F*_(1,11)_ = 23.762, *β* = 0.827, *p* < 0.001].

**Table 1:** Summary of the second set of stepwise regression analyses regarding neuropsychological tests with the greatest contribution to each functional domain, as assessed by the DAD ratings.

**Dependent Variable**	**Predictive Factor**	**B**	**SE B**	**Standardized *β***	***R^2^***	***F***	**df**	***p***
ADLs	FES working memory	23.568	4.835	0.827	0.684	23.762	1, 11	<0.001
BADLs	FES working memory	20.461	5.824	0.727	0.529	12.344	1, 11	0.005
IADLs	FES working memory	25.917	5.948	0.796	0.633	18.985	1, 11	0.001
housework and leisure activities	dot counting	2.808	0.925	0.675	0.456	9.208	1, 11	0.011
finance and correspondence	list learning-immediate recall	4.254	0.928	0.810	0.656	21.014	1, 11	0.001
outing	list learning-recognition	6.407	1.531	0.784	0.614	17.511	1, 11	0.002
meal preparation	CPT performance-errors	−30.833	11.939	−0.614	0.377	6.670	1, 11	0.025
telephoning	FES working memory	38.084	7.487	0.838	0.702	25.875	1, 11	0.001
hygiene	FES working memory	28.629	8.899	0.696	0.485	10.349	1, 11	0.008

CPT performance-errors: the total number of trials in the Continuous Performance test where the subject provided more than one response. SE, Standard Error; df, degrees of freedom.

Furthermore, it was found that the working memory subtest of the FES, the total score of the FES and the phonemic verbal fluency test contributed significantly to BADL-score, as assessed by the DAD ratings. In the second set of stepwise regression analyses, it was found that again the working memory subtest of the FES had the greatest unique contribution to BADL performance, accounting for 52.9% of variance in BADL functioning [*F*_(1,11)_ = 12.344, *β* = 0.727, *p* = 0.005].

With respect to IADLs, it was found that the working memory subtest of the FES, the FES total score and the recognition task of the list learning test contributed significantly to IADL-score. In the second set of stepwise regression analyses, it was found that again the working memory subtest of the FES had the greatest contribution to IADLs, accounting for 63.3% of variance in IADL functioning [*F*_(1,11)_ = 18.985, *β* = 0.796, *p* = 0.001]. In all the aforementioned results, higher scores in the neuropsychological tests were associated with higher ratings on the ADLs and *vice versa* (For the shake of conciseness, in the rest of the section only the final results from the second sets of stepwise regression analyses will be presented. The tables including information for both sets of analyses are located in the Supplementary Material of the present study).

As shown in Table 1, in terms of specific domains of everyday functioning, the dot counting test was the only neuropsychological measure that contributed significantly to performance on housework and leisure activities [*R^2^* = 0.456, *F*_(1,11)_ = 9.208, *β* = 0.675, *p* = 0.011]. Furthermore, it was found that the immediate recall of the list learning test had the greatest contribution to finance and correspondence management [*R^2^* = 0.656, *F*_(1,11)_ = 21.014, *β* = 0.810, *p* = 0.001], the recognition task of the list learning test had the greatest contribution to the ability to go on an outing [*R^2^* = 0.614, *F*_(1,11)_ = 17.511, *β* = 0.784, *p* = 0.002], and the working memory subtest of the FES had the greatest contribution to telephoning [*R^2^* = 0.702, *F*_(1,11)_ = 25.875, *β* = 0.838, *p* = 0.001]. Moreover, a variable related to the total number of trials where the participants mistakenly provided more than one response in the Continuous Performance test (“CPT performance-errors”) had the greatest contribution to the ability to prepare a meal [*R^2^* = 0.377, *F*_(1,11)_ = 6.670, *β* = −0.614, *p* = 0.025]. In addition, the working subtest of the FES had the greatest contribution to the ability to take care of personal hygiene [R^2^ = 0.485, *F*_(1,11)_ = 10.349, *β* = 0.696, *p* = 0.008].

Finally, as revealed by Table 2, results showed that a variable related to the total number of non-target trials in the CPT test where the subjects correctly did not respond (“CPT non-target, correct answers”) and the recognition task of the list learning test had the greatest contribution to medication management. More specifically, in the first step of the stepwise model that resulted, “CPT non-target, correct answers” entered as a significant predictor [*R^2^* = 0.552, *F*_(1,11)_ = 13.558, *β* = 0.743, *p* = 0.004]. In the second step, the score of the recognition task of the list learning test also entered in the model (*β* = 0.462, *p* = 0.005), accounting for 74.4% of variance in medication adherence [*R^2^* = 0.744, *F*_(2, 10)_ = 14.565, *p* = 0.001]. See Supplementary Material for a summary of the first and second sets of stepwise regression analyses regarding the cognitive correlates of functional status in FTD patients.

**Table 2:** Summary of the second set of stepwise regression analyses regarding neuropsychological tests with the greatest contribution to medication adherence, as assessed by the DAD ratings.

**Dependent Variable**	**Steps**	**Predictive Factor**	**B**	**SE B**	**Standardized *β***	***R^2^***	***F***	**df**	***p***
**Medication**	1	CPT non-target, correct	4.481	1.217	0.743	0.552	13.558	1, 11	0.004
	2	CPT non-target, correct	3.606	1.016	0.598	0.744	14.565	2, 10	0.001
		list learning-recognition	5.344	1.948	0.462				

CPT non-target, correct: the total number of non-target trials in the Continuous Performance test where the subject correctly did not respond. SE, Standard Error; df, degrees of freedom.

### Cognitive correlates of behavioral disturbances in FTD

With respect to cognitive factors that are associated with behavioral symptoms in FTD, results showed that the word-condition of the Stroop test had the greatest contribution to logopenia, as assessed by the FBI ratings [*R^2^* = 0.540, *F*_(1,11)_ = 12.909, *β* = −0.735, *p* = 0.004] (see Table 3). Furthermore, the immediate recall from the story memory test had the greatest contribution to inattention [*R^2^* = 0.702, *F*_(1,11)_ = 25.875, *β* = 0.784, *p* = 0.001] and the mean response time of the correct shifted trials in the shift block of the Set-Shifting test had the greatest contribution to obsessions [*R^2^* = 0.566, *F*_(1,11)_ = 5.178, *β* =0.784, *p* = 0.044].

**Table 3:** Summary of the second set of stepwise regression analyses regarding the neuropsychological tests with the greatest contribution to each behavioral symptom, as assessed by the FBI ratings.

**Dependent Variable**	**Predictive Factor**	***B***	**SE B**	**Standardized *β***	***R^2^***	***F***	**df**	***p***
logopenia	Stroop-words	−0.028	0.008	−0.735	0.540	12.909	1, 11	0.004
inattention	story memory-immediate recall	−0.087	0.034	−0.606	0.367	6.373	1, 11	0.028
obsessions	SS shifted-mean	0.196	0.086	0.566	0.320	5.178	1, 11	0.044
loss of insight	story memory-delayed recall	−0.270	0.056	−0.831	0.691	24.588	1, 11	<0.001
Inflexibility	Stroop-interference index	0.114	0.048	0.581	0.337	5.597	1, 11	0.037

df, degrees of freedom; SE, Standard Error.

Moreover, the interference index from the Stroop test was found to have the greatest contribution to inflexibility [*R^2^* = 0.337, *F*_(1,11)_ = 5.597, *β* = 0.581, *p* = 0.037] and the delayed recall of the story memory test was found to have the greatest contribution to loss of insight [*R^2^* = 0.691, *F*_(1,11)_ = 24.588, *β* = −0.831, *p* < 0.001]. Finally, it was found that none of the neuropsychological performances contributed significantly to apathy, aspontaneity, and irritability. See Supplementary Material for a summary of the first and second sets of stepwise regression analyses regarding the cognitive correlates of behavioral disturbances in FTD patients.

### Behavioral correlates of functional status in FTD

With regards to behavioral symptoms that are associated with functional outcomes in FTD patients, results showed that personal neglect along with the sub-score of negative symptoms, as assessed by the FBI, had the greatest contribution to the total ADL ratings. More specifically, in the first step of the two-step model that resulted, personal neglect entered as a significant predictor [*R^2^* = 0.673, *F*_(1,11)_ = 22.593, *β* = −0.820, *p* = 0.001]. In the second step, the sub-score of negative symptoms also entered in the model (*β* = −0.453, *p* = 0.026), accounting for 80.5% of variance in ADL performance [*R^2^* = 0.805, *F*_(2, 10)_ = 20.608, *p* < 0.001]. Furthermore, personal neglect was found to have the greatest contribution to BADLs [*R^2^* = 0.666, *F*_(1,11)_ = 21.890, *β* = −0.816, *p* = 0.001], whereas the sub-score of negative symptoms was found to have the greatest contribution to IADLs [R^2^ = 0.693, *F*_(1,11)_ = 24.785, *β* = −0.832, *p* < 0.001].

When it comes to each functional domain separately, personal neglect was found to have the greatest contribution to hygiene [*R^2^* = 0.653, *F*_(1,11)_ = 20.727, *β* = −0.808, *p* = 0.001] and telephoning [*R^2^* = 0.358, *F*_(1,11)_ = 6.122, *β* = −0.598, *p* = 0.031]. Moreover, the sub-score of negative symptoms had the greatest contribution to the three following domains: going on an outing [*R^2^* = 0.772, *F*_(1,11)_ = 37.290, *β* = −0.879, *p* < 0.001], managing finance and correspondence [*R^2^* = 0.720, *F*_(1,11)_ = 28.333, *β* = −0.849, *p* < 0.001], and taking care of medication [*R^2^* = 0.428, *F*_(1,11)_ = 8.230, *β* = −0.654, *p* = 0.015]. In addition, aspontaneity was found to have the greatest contribution to the ability to prepare a meal [*R^2^* = 0.381, *F*_(1,11)_ = 6.785, *β* = −0.618, *p* = 0.024].

Finally, as illustrated by Table 4, in the matter of household chores and recreational activities, a two-step model resulted from the analyses. More specifically, in the first step of the model apathy entered as significant predictor [*R^2^* = 0.859, *F*_(1,11)_ = 31.054, *β* = −0.859, *p* < 0.001]. In the second step, loss of insight also entered in the model (*β* = −0.316, *p* = 0.048), accounting for 90.9% of variance [*R^2^* = 0.909, *F*_(1,11)_ = 23.764, *p* < 0.001]. Higher scores on behavioral symptoms (as assessed by the FBI) were associated with lower scores on the ADLs (as assessed by the DAD), and* vice versa*. See Supplementary Material for a summary of the first and second sets of stepwise regression analyses regarding the behavioral correlates of functional status in FTD.

**Table 4:** Summary of the second set of stepwise regression analyses regarding the behavioral symptoms with the greatest contribution to housework and leisure activities, as assessed by the FBI and DAD ratings.

**Dependent Variable**	**Steps**	**Predictive Factor**	**B**	**SE B**	**Standardized *β***	***R^2^***	***F***	**df**	***p***
housework and leisure activities	1	apathy	−19.524	3.504	−0.859	0.738	31.054	1, 11	<0.001
	2	apathy,	−17.011	3.197	−0.316	0.826	23.764	2, 10	<0.001
		loss of insight	−5.173	2.302				

df, degrees of freedom; SE, Standard Error.

### Cognitive correlates of compensatory strategy use

The performance on the following neuropsychological tests were found to have a significant contribution to the use of compensatory strategies among FTD patients: (1) the backward task of the digit span test; (2) the variable regarding the total number of incongruent trials in the Flanker test where subjects’ responses were correct (“FL incongruent-correct”); (3) the part B of the TMT; (4) the semantic verbal fluency test; (5) the FES total score; (6) the word-condition of the Stroop test; (7) the immediate recall of the list learning test; (8) the copy task; (9) the delayed recall from the Taylor complex figure test and, finally; and (10) the confrontation naming test.

As shown in Table 5, among these neuropsychological measures, the immediate recall of the list learning test, as well as the backward task of the digit span test were found to have the greatest contribution to the ability to use compensatory strategies. More specifically, in the first step of the model that resulted from the stepwise regression analysis, the immediate recall of the list learning test entered as a significant predictor [*R^2^* = 0.617, *F*_(1,11)_ = 17.717, *β* = 0.785, *p* = 0.001]. In the second step, the backward task of the digit span test also entered in the model (*β* = 0.570, *p* = 0.010), accounting for 80.9% of variance in compensatory strategy use [*R^2^* = 0.809, *F*_(2, 10)_ = 21.139, *p* < 0.001].

**Table 5:** Summary of the second set of stepwise regression analyses regarding the neuropsychological tests with the greatest contribution to compensatory strategy use, as assessed by the IADL-C ratings.

**Dependent Variable**	**Steps**	**Predictive Factor**	**B**	**SE B**	**Standardized *β***	***R^2^***	***F***	**df**	***p***
**compensatory strategy use**	1	list learning-immediate recall	0.485	0.115	0.785	0.617	17.717	1, 11	0.001
	2	list leaning-immediate recall,	0.352	0.095	0.570	0.809	21.139	2, 10	<0.001
		backward digit span	4.465	0.463	0.488				

SE, Standard Error; df, degrees of freedom.

## Discussion

The results of the present study delineate the profile of behavioral disturbances and functional limitations that patients with FTD encounter. First, apathy was found to be the most prevalent behavioral symptom in FTD, since the vast majority of knowledgeable informants (approximately 85%) reported it as existing to a moderate or severe level among patients. Apathy is associated with poor treatment outcomes and decreased level of everyday functioning (Clarke et al., 2008). Given the prevalence of this syndrome among FTD patients and its devastating effect on functional status, further efforts to gain a better understanding of its pathogenesis and its neural, clinical, and sociodemographic correlates are necessitated (Clarke et al., 2008).

Interestingly, negative symptoms seem to predominate in the clinical presentation of FTD patients. Indeed, as previous studies have indicated, negative symptoms are a core component of FTD (Boone et al., 2003; Benussi et al., 2020). It has also been suggested that FTD patients are three times more likely to demonstrate negative symptoms than individuals with AD (Boone et al., 2003).

Furthermore, results indicated that FTD patients also display marked positive symptoms. In fact, obsessions and irritability were found to be the most frequent positive behavioral manifestations, being present in approximately 62% of the patients. This finding regarding obsessions replicated findings from previous studies, suggesting that the presence of obsessive/repetitive behaviors is characteristic of FTD, and especially bvFTD cases. Patients with bvFTD often demonstrate perseverative, stereotyped or compulsive-ritualistic behaviors as an early manifestation of their disorder (Moheb et al., 2019). Irritability has also been reported as a common neuropsychiatric symptom and often as a pre-dementia risk marker (Ismail et al., 2018).

This study also provided significant information regarding the functional areas where FTD patients encounter the greater difficulties. Engaging in leisure activities and doing the household chores constitute the functional domains where FTD patients demonstrate most prominent difficulties. A plausible explanation is that both recreational activities and household affairs rely on individual’s motivation and self-initiated behavior. However, as mentioned above, patients with FTD display high levels of apathy that may undermine their ability to engage in such activities.

Managing the finance and correspondence, as well as going on an outing are the next more impaired functional domains. Patients also encounter marked difficulties with telephoning, meal preparation, and medication management. These findings are consistent with previous studies, indicating that FTD patients face significant difficulties in various IADL tasks (Giebel et al., 2021). On the other hand, activities that are important for survival and self-care, such as dressing, eating and continence, seem to be preserved and only performance on hygiene was relatively lower than the other BADL activities.

Overall, it seems that IADLs are much more impaired than BADLs in FTD patients. This finding is consistent with previous research which has shown that FTD initially affects IADLs, while decline in BADLs develops in the later stages of the disease (Mioshi et al., 2010). The functional deficits seen in these patients have significant implications, since they are associated with higher levels of dependence and, consequently, higher levels of caregiver distress and frustration (Moheb et al., 2017).

The extensive assessment of behavioral disturbances and daily functioning restrictions in dementia is considered of high importance for many reasons. First, behavioral symptoms and performance on activities of daily living can help clinicians determine the severity of disease and, therefore, the level of functional dependence and care demands of these patients. In addition to this, everyday functioning profiles can contribute to the differential diagnosis of the disease, because different dementia syndromes display different profiles of functional deficits (Giebel et al., 2021). Furthermore, knowledge about the domains of daily living where these patients demonstrate the most profound difficulties, can be integrated in the effective care planning of everyday functioning, and can provide clinicians with valuable guidelines for the design of rehabilitation programs (Giebel et al., 2021).

With respect to cognitive factors that are associated with functional outcomes, results suggested that working memory constitutes the strongest cognitive correlate of functional status in FTD patients. The magnitude of variance in everyday functioning explained by working memory reached up to 68.4%. Among other neuropsychological performances, working memory emerged as the cognitive domain with the greatest contribution not only to general ADLs, but also to BADLs and IADLs. This finding is in line with previous studies indicating that measures of executive cognition and especially working memory are associated with ratings of everyday functioning in patients with neurocognitive disorders, such as MCI or AD (Aretouli and Brandt, 2010; Pillai et al., 2014). Similarly, longitudinal studies have shown that reservation of working memory relates to a slower rate of functional decline, whereas working memory impairment seems to have a pernicious effect on functional outcomes (Pillai et al., 2014). The specific mechanisms whereby working memory affects everyday functioning have not been yet completely identified and warrant further investigation. However, it can be understood that numerous everyday activities depend on cognitive processes that involve active maintenance of specific elements in working memory, while other elements are being processed (Humphrey et al., 2001). Additionally, several other cognitive abilities essential for everyday functioning rely on working memory, such as the ability to monitor conflicts between the actual and the required sequence of actions (Shallice and Burgess, 1996).

With reference to the remaining functional domains, as assessed by the DAD, results showed that management of finance and correspondence was associated with many neuropsychological measures. This probably happens because finance management constitutes a cognitively demanding functional domain and, therefore, entails several cognitive components. Among them, a measure of short-term verbal memory was found to be the strongest cognitive correlate of financial capacity. Short-term verbal memory depends on attentional abilities and immediate information storage. It can be understood that the ability to retain information over short periods of time is critical in mental arithmetic that goes with money management (Raghubar et al., 2010).

Medication adherence was predicted by inhibitory control and recognition of verbal information. This finding agrees with previous studies indicating that the performance on memory and executive function measures can be used to identify older adults who may be at risk for failure to take medicines as prescribed (Insel et al., 2006). Indeed, taking medication consistently relies on the recruitment of cognitive processes that involve both memory and executive cognition. On the one hand, adhering to medication relies on encoding and storage of information about the medicine (e.g., why does the person need to take the medicine, why is it important), as well as instructions regarding the conditions under which the medicine is taken (e.g., when and how; Insel et al., 2006). On the other hand, taking medicines depends also on intention retrieval, initiation, inhibition of environmental distractions, tolerance with possible delays until the conditions are appropriate, execution of the action and, finally, self-monitoring regarding whether the medicine was taken as intended (e.g., at the correct time, according to the right dosage, etc.; Insel et al., 2006). Given the critical role of compliance with treatment regimen in slowing dementia’s progression, the findings of the present study are of great importance, because they imply that cognitive rehabilitation focused on the aforementioned cognitive domains may have the potential to improve medication management.

Meal preparation was predicted by a measure of inhibitory control. Meal preparation constitutes a complex multi-step task that depends significantly on executive cognition. It relies on the recognition of the need to prepare a meal (initiation), planning and organization of the required ingredients and cookware and, finally, successful completion of the action in a safe and acceptable manner. Finally, going on an outing was found to be predicted by a measure of long-term memory that relies on memory storage and recognition of verbal information. Going out is also a complex everyday activity that involves multiple steps, such as taking the initiative to undertake an outing, organizing the outing with respect to outfit, weather, keys, necessary money, and transportation and, finally, returning from the destination with the appropriate items (e.g., shops) and without getting lost. It is clear that in the aforementioned steps, memory components play a crucial role and determine the extent to which performance will be effective.

Results regarding the cognitive factors that are associated with the presence of behavioral disturbances among FTD patients showed that apathy, aspontaneity and irritability are not explained by any neuropsychological performance. This finding indicates that the aforementioned behavioral symptoms, that are prominent in FTD, probably are not characterized by any cognitive component, but instead represent separate clinical manifestations that are mediated by different neural systems and neuropathological mechanisms. This finding is considered crucial for the better understanding and, thus treatment, of these behavioral manifestations and implies that apathy, aspontaneity and irritability may not be alleviated with the implementation of cognitive rehabilitation therapies, but instead with other therapeutical interventions (such as, pharmacological management, psychosocial interventions or psychotherapy). Given the high prevalence of these symptoms across the dementias (Clarke et al., 2008), as well as their devastating impact on functional status (Moheb et al., 2017), this finding renders the investigation of new, effective non-cognitive interventions for the management of these clinical manifestations as imperative.

On the other hand, results showed that some of the most prominent behavioral symptoms among FTD patients were predicted by neuropsychological performances. More specifically, logopenia was found to be predicted by the word-condition of the Stroop test, which is a task that relies on language abilities. This finding implies that tasks that require word production (even word reading) can predict if an individual exhibits decreased amount of speech in everyday life. Furthermore, inattention appeared to be predicted by the immediate recall of the story memory test, which indeed constitutes a task that relies mostly on attentional abilities and short-term verbal memory. Therefore, this task can possibly be used by clinicians in order to reflect if patients demonstrate attention deficits in real-life performance.

Inflexibility in everyday tasks was predicted by the interference index of the Stroop test, which indicates that deficits in executive functions, such as inhibitory control, can probably explain the rigid and inflexible way of thinking that FTD patients tend to demonstrate in everyday life. Loss of insight was found to be predicted by the delayed recall of the story memory test. This finding suggests that impairment in the long-term memory may contribute to the anosoagnosia and lack of insight that FTD patients often exhibit concerning their cognitive problems and changes in behavior. Finally, obsessions were found to be predicted by a variable related to the set-shifting test. It is noteworthy that set-shifting is the ability to switch between different mental tasks and is related to cognitive flexibility (Miyake and Friedman, 2012). The finding of the present study regarding the cognitive component of obsessions is consistent with previous studies suggesting that cognitive flexibility is significantly impaired in patients with disorders, such as obsessive-compulsive disorder (OCD; Gruner and Pittenger, 2017) and such deficits may contribute to obsession-related symptomatology.

With regards to the contribution of behavioral disturbances to functional impairment among FTD patients, results revealed that negative symptoms account for a large proportion of variance in everyday functioning (ADLs). In addition, personal neglect was found to be the strongest behavioral correlate of performance on BADLs (such as, hygiene), while negative symptoms in general were found to be the strongest correlate of performance on IADLs. Besides, negative symptoms were associated with performance on specific IADL tasks, such as managing finance and correspondence, taking medication, and going on outings.

Apathy and loss of insight were found to be the strongest behavioral correlates of performance on housework and leisure activities. Indeed, individuals that demonstrate apathy and, therefore, a loss of interest in being involved in previously rewarding activities or in maintaining social connections, are less likely to engage in recreational or other similar activities. Moreover, loss of insight may have an additional negative impact, since individuals who are not aware of their problems probably cannot recognize the need for engagement in such activities. Finally, the ability to prepare effectively a meal was associated with aspontaneity, that is, a lack of spontaneous behavior. It is clear that individuals who display low levels of self-initiated behavior, are less likely to take the initiative to undertake the preparation of a meal, without reminder or encouragement. The above findings imply that functional impairment, which historically has been attributed mostly to cognitive deficits, can be also explained by behavioral symptomatology (Reichman and Negron, 2001).

With respect to our last research goal, we investigated the cognitive factors that are associated with the use of compensatory strategies among FTD patients. Both short-term verbal memory and working memory were predictive of the application of compensatory strategies in activities of daily living. Taken into consideration that compensatory strategy use (such as assistive technology, external reminders, and environmental cues) can support remarkably IADL performance and increase patients’ functional independence, this finding is considered of high importance. It implies that cognitive rehabilitation programs that focus on these cognitive domains probably have the potential to enhance individuals’ readiness to use such strategies and, therefore, help them overcome or mitigate cognitive challenges that may arise when performing IADL tasks.

Previous studies have shown that training in the use of compensatory strategies may contribute to greater resilience and higher levels of functioning in daily life, even in the face of cognitive decline (Tomaszewski Farias et al., 2018). Therefore, it is suggested that future studies focus on the development of cognitive interventions that directly target such strategies. Indeed, effective non-pharmacological cognitive interventions to enhance the functional independence of patients with dementia are an urgent international priority (Gates et al., 2011). In addition, it is recommended that future studies and clinical trials include in their protocols the use of psychometric tools that assess in a quantified way the use of compensatory strategies in patients with dementia. Information about the frequency and type of compensatory strategies employed, can also function as a criterion for the assessment of effectiveness of interventions applied to these patients.

To conclude, our findings confirm and extent previous reports that executive dysfunction and—to a lesser extent—memory deficits, as well as negative (behavioral) symptoms can predict the severity of everyday functional disability among FTD patients. Adopting an approach towards the identification of cognitive and behavioral factors that are associated with functional impairment in FTD is of high importance and has significant scientific as well as clinical implications to physicians, patients, and their families. Such knowledge will allow improved prognostic estimates in a disease that affects predominantly young populations and, additionally, will contribute to the design of interventions that, if implemented early, may have the potential to improve functional outcomes and delay the rate of functional decline among FTD patients (Josephs et al., 2011; Moheb et al., 2017).

In addition, investigating the correlations between cognitive performances and measures of everyday functioning will also contribute to the evaluation of ecological validity of a wide range of neuropsychological tests. Evaluating the ecological validity of neuropsychological tests has become an increasingly important topic over the past decade (Chaytor and Schmitter-Edgecombe, 2003). In the field of clinical neuropsychology, this knowledge can be very useful and may help clinicians gather important information regarding their patients’ real-world performances.

Finally, it should be noted that the empirical results reported herein have to be considered in the light of some limitations. First, the findings of the present study are considered preliminary, due to the quite small sample size included. The fact that several explanatory variables were involved in the regression analyses of a small sample size constitutes an important limitation that affects the validity of our findings. However, it is worth mentioning that frontotemporal dementia is a clinical syndrome with relatively low prevalence (15–22/100,000 persons; Onyike and Diehl-Schmid, 2013), therefore identifying patients who meet the inclusion criteria is challenging. An additional difficulty was the very extensive and thorough assessment of each patient and their caregiver which significantly increased the time required to collect the data for each study participant. Nevertheless, we conducted this pilot study without reducing the number of administered procedures to explore the potential connections between cognition, behavior, and everyday functioning in this clinical group. The results of the present study are intended to provide only preliminary associations, which need to be confirmed by future studies with much larger samples. In addition, the fact that our FTD sample is a non-random sample of convenience affects the generalizability of the study’s results.

Another limitation is the cross-sectional nature of our analyses, which restricted some of our interpretations. Ideally, in the future, longitudinal designs are required in order to gain a better understanding of the evolving profile of functional impairment in relationship to cognitive and behavioral measures. Furthermore, one might argue that informant-based reports used to assess everyday functioning are less valid than performance-based tests. However, performance of an activity in a structured clinic or lab setting may not reflect accurately the typical performance of patients in everyday life and, for that reason, indirect measures of functional abilities were preferred. Last but not least, the FTD sample of the present study is heterogeneous, since it is consisted of individuals of all the three clinical FTD subtypes. However, analyses were performed to all FTD patients as a group and not to each subgroup separately, mainly due to small subgroup sample sizes. Given the remarkable differences in cognitive and behavioral deficits between FTD subgroups and the subsequent different functional deficits, further investigation of functional impairment within these subgroups is warranted.

## Data availability statement

The raw data supporting the conclusions of this article will be made available by the authors, without undue reservation.

## Ethics statement

The studies involving human participants were reviewed and approved by The Bioethics and Ethics Committee of Medical School of Aristotle University of Thessaloniki, Greece. The patients/participants provided their written informed consent to participate in this study.

## Author contributions

EC: collected the data, analyzed the data, and wrote this article. PI: supervised the recruitment and clinical examination of the patients. DM: provided feedback on drafts of the manuscript and approved the final draft. EK: provided guidance on the administration and data analysis of the “NIH-EXAMINER” battery for the assessment of executive functions. EA: provided supervision and guidance through each stage of the study regarding psychometric tool selection, data collection and analysis, as well as interpretation of the findings. All authors contributed to the article and approved the submitted version.
