# Supplementary material for: The cognitive and behavioral correlates of functional status in patients with frontotemporal dementia: A pilot study
Source: Front Hum Neurosci. 2023 Feb 22;17:1087765. doi: 10.3389/fnhum.2023.1087765 (PMC10009888; doi:10.3389/fnhum.2023.1087765)
Supplement: Supplementary file 1 [file Data_Sheet_1.pdf]

Appendix

Table 1: Demographic Information and Disease Duration of FTD participants

FTD patients	
**n**	13 (bvFTD=9; nfvPPA=3; svPPA=1)
**Gender, % male**	61,50%
**Age, years**	67 (9,13)
**Education, years**	12,46 (4,67)
**Duration of disease, years**	3,38 (1,71)

Parentheses denote standard deviation; bvFTD, behavioral variant frontotemporal dementia; nfvPPA, nonfluent variant primary progressive aphasia; svPPA, semantic variant primary progressive aphasia.

Table 2: Summary of the neuropsychological tests administered for the assessment of each cognitive domain.

Cognitive Domain	Tests
Working Memory/Updating	1. **Digit Span Test** (Kosmidis et al., 2011; Weschler, 1997)<br>2. **Dot Counting Test** (Kramer et al., 2014)<br>3. **N-Back Test** (Kramer et al., 2014)
Set-Shifting	1. **Set Shifting Test** (Kramer et al., 2014)<br>2. **Trail Making Test-Part B (TMT)** (Reitan, 1958; Vlachou & Kosmidis, 2002)
Inhibition	1. **Flanker Test** (Kramer et al., 2014)<br>2. **Continuous Performance Test (CPT)** (Kramer et al., 2014)<br>3. **Stroop Test-words and colors** (Stroop, 1935)
Verbal Fluency	1. **Semantic/Category Verbal Fluency Test** (Kosmidis et al., 2011)<br>2. **Phonemic Verbal Fluency Test** (Kosmidis et al., 2011)
Processing (Visuo-Motor) Speed	1. **Trail Making Test-Part A (TMT)** (Reitan, 1958; Vlachou & Kosmidis, 2002)<br>2. **Stroop Test-words** (Stroop, 1935)<br>3. **Stroop Test-colors** (Stroop, 1935)
General Executive Functioning	1. **FRONTIER Executive Screen (FES)** (Leslie et al., 2016)
Verbal Memory	1. **Story Memory Test** (Kosmidis et al., 2011)<br>2. **Word Learning Test** (Kosmidis et al., 2011)
Visual Memory	**Taylor Complex Figure Test** (Taylor, 1959)
Visuospatial Abilities	**Taylor Complex Figure Test-copy** (Taylor, 1959)
Language	**Confrontation Naming Test** (Kaplan et al., 2001; Kosmidis et al., 2011)

Table 3: Summary of the first set of stepwise regression analyses regarding the contribution of neuropsychological tests to functional status, as assessed by the DAD ratings.

Dependent Variable	Predictive Factor	*B*	SE B	Standardized *β*	R^2	*F*	df	*p*
**ADLs**	FES working memory	23.568	4.835	0.827	0.684	23.762	1.11	<.001
	FES total score	6.953	2.143	0.699	0.489	10.530	1.11	0.008
	phonemic verbal fluency	4.895	10.679	0.630	0.397	7.256	1.11	0.21
	list learning-recognition	4.868	1.433	0.716	0.512	11.547	1.11	0.006
**BADLs**	FES working memory	20.461	5.824	0.727	0.529	12.344	1.11	0.005
	FES total score	6.928	2.096	0.706	0.489	10.928	1.11	0.007
	phonemic verbal fluency	5.115	1.721	0.667	0.445	8.837	1.11	0.013
**IADLs**	FES working memory	25.917	5.948	0.796	0.633	18.985	1.11	0.001
	FES total score	7	2.698	0.616	0.380	6.731	1.11	0.025
	list learning-recognition	5.990	1.493	0.771	0.594	16.087	1.11	0.002
**housework and leisure activities**	dot counting	2.808	0.925	0.675	0.456	9.208	1.11	0.011
**finance and correspondence**	dot counting	5.484	1.492	0.743	0.551	13.571	1.11	0.004
	TMT-Part B	-0.203	0.080	-0.609	0.370	6.468	1.11	0.027
	FES total score	9.834	4.050	0.591	0.349	5.897	1.11	0.034
	Stroop-colors	1.817	0.674	0.631	0.398	7.281	1.11	0.021
	list learning-immediate recall	4.254	0.928	0.810	0.656	21.014	1.11	0.001
	list learning-recognition	8.893	2.145	0.781	0.610	17.185	1.11	0.002
	Taylor complex figure test-delayed recall	5.749	2.174	0.623	0.389	6.993	1.11	0.023
	confrontation naming test	2.280	0.990	0.571	0.326	5.309	1.11	0.042

**outing**	dot counting	3.674	1.152	0.693	0.480	10.166	1.11	0.009
	list learning-recognition	6.407	1.531	0.784	0.614	17.511	1.11	0.002
**telephoning**	working memory FES	38.084	7.487	0.838	0.702	25.875	1.11	<0.001
	FL incongruent-correct	2.382	0.652	0.741	0.549	13.369	1.11	0.004
	verbal fluency phonemic	7.567	2.955	0.611	0.373	6.557	1.11	0.026
	Stroop-words	0.974	0.419	0.574	0.330	5.418	1.11	0.040
**meal preparation**	CPT performance-errors	-30.833	11.939	-0.614	0.377	6.670	1.11	0.025
**medication**	FES working memory	33.000	10.707	0.681	0.463	9.500	1.11	0.010
	FES total score	12.044	3.578	0.712	0.507	11.329	1.11	0.006
	verbal fluency phonemic	8.234	3.112	0.624	0.389	7.002	1.11	0.023
	CPT non-target, correct	4.481	1.217	0.743	0.552	13.558	1.11	0.004
	list learning-recognition	7.517	2.651	0.650	0.422	8.039	1.11	0.016
**hygiene**	FES working memory	28.629	8.899	0.696	0.485	10.349	1.11	0.008
	verbal fluency phonemic	7.249	2.574	0.647	0.419	7.932	1.11	0.017

FL incongruent-correct: the total number of incongruent trials in the Flanker test where the subject's response was correct (incongruent are the trials where the non-target arrows point in the opposite direction than the centrally presented target arrow),

CPT performance-errors: the total number of trials in the Continuous Performance test where the subject incorrectly provided more than one response,

CPT non-target, correct: the total number of non-target trials in the Continuous Performance test where the subject correctly did not respond,

SE: Standard Error,

df: degrees of freedom

Table 4: Summary of the second set of stepwise regression analyses regarding neuropsychological tests with the greatest contribution to each functional domain, as assessed by the DAD ratings.

Dependent Variable	Predictive Factor	B	SE B	Standardized β	R^2	F	df	p
**ADLs**	FES working memory	23.568	4.835	0.827	0.684	23.762	1,11	<0.001
**BADLs**	FES working memory	20.461	5.824	0.727	0.529	12.344	1,11	0.005
**IADLs**	FES working memory	25.917	5.948	0.796	0.633	18.985	1,11	0.001
**housework and leisure activities**	dot counting	2.808	0.925	0.675	0.456	9.208	1,11	0.011
**finance and correspondence**	list learning-immediate recall	4.254	0.928	0.810	0.656	21.014	1,11	0.001
**outing**	list learning-recognition	6.407	1.531	0.784	0.614	17.511	1,11	0.002
**meal preparation**	CPT performance-errors	-30.833	11.939	-0.614	0.377	6.670	1,11	0.025
**telephoning**	FES working memory	38.084	7.487	0.838	0.702	25.875	1,11	0.001
**hygiene**	FES working memory	28.629	8.899	0.696	0.485	10.349	1,11	0.008

CPT performance-errors: the total number of trials in the Continuous Performance test where the subject provided more than one response, SE: Standard Error, df: degrees of freedom

Table 5: Summary of the second set of stepwise regression analyses regarding neuropsychological tests with the greatest contribution to medication adherence, as assessed by the DAD ratings.

**Dependent Variable**	**Steps**	**Predictive Factor**	**B**	**SE B**	**Standardized *β***	R^2	***F***	**df**	***p***
**Medication**	1	CPT non-target, correct	4.481	1.217	0.743	0.552	13.558	1, 11	0.004
	2	CPT non-target, correct	3.606	1.016	0.598	0.744	14.565	2, 10	0.001
		list learning-recognition	5.344	1.948	0.462				

CPT non-target, correct: the total number of non-target trials in the Continuous Performance test where the subject correctly did not respond, SE: Standard Error, df: degrees of freedom

Table 6: Summary of the first set of stepwise regression analyses regarding the contribution of neuropsychological tests to behavioral disturbances, as assessed by the FBI ratings.

Dependent Variable	Predictive Factor	*B*	SE B	Standardized *β*	R^2	*F*	df	*p*
logopenia	Stroop-words	-0.028	0.008	-0.735	0.540	12.909	1,11	0.004
	FL total-correct	-0.21	0.009	-0.564	0.314	5.143	1,11	0.044
inattention	story memory-immediate recall	-0.087	0.034	-0.606	0.367	6.373	1,11	0.028
obsessions	SS shifted-mean	0.196	0.086	0.566	0.320	5.178	1,11	0.044
loss of insight	Taylor-recognition	-0.367	0.105	-0.724	0.525	12.136	1,11	0.005
	story memory-delayed	-0.270	0.056	-0.831	0.691	24.588	1,11	<0.001
	FES total score	-0.350	0.137	-0.611	0.374	6.564	1,11	0.026
	FES verbal fluency	-0.471	0.188	-0.602	0.363	6.256	1,11	0.029
	dot counting	-0.183	0.053	-0.720	0.519	11.871	1,11	0.005
inflexibility	Stroop-interference index	0.114	0.048	0.581	0.337	5.597	1,11	0.037

FL total-correct: the total number of trials of both congruent and incongruent conditions of the Flanker test where the subject's response was correct,

SS shifted-mean: the mean response time of the correct shifted trials in the shift block of the Set Shifting test,

SE: Standard Error, df: degrees of freedom

Table 7: Summary of the second set of stepwise regression analyses regarding the neuropsychological tests with the greatest contribution to each behavioral symptom, as assessed by the FBI ratings.

**Dependent Variable**	**Predictive Factor**	***B***	**SE B**	**Standardized *β***	***R^2***	***F***	**df**	***p***
logopenia	Stroop-words	-0.028	0.008	-0.735	0.540	12.909	1,11	0.004
inattention	story memory-immediate recall	-0.087	0.034	-0.606	0.367	6.373	1,11	0.028
obsessions	SS shifted-mean	0.196	0.086	0.566	0.320	5.178	1,11	0.044
loss of insight	story memory-delayed recall	-0.270	0.056	-0.831	0.691	24.588	1,11	<0.001
Inflexibility	Stroop-interference index	0.114	0.048	0.581	0.337	5.597	1,11	0.037

SS shifted-mean: the mean response time of the correct shifted trials in the shift block of the Set Shifting test,

SE: Standard Error, df: degrees of freedom

Table 8: Summary of the first set of stepwise regression analyses regarding the contribution of behavioral symptomatology to functional status, as assessed by the FBI and DAD ratings, respectively.

Dependent Variable	Predictive Factor	B	SE B	Standardized β	R^2	*F*	df	*p*
**BADLs**	negative symptoms	-1.933	0.834	-0.573	0.328	5.373	1,11	0.041
	positive Symptoms	-2.223	0.918	-0.589	0.347	5.857	1,11	0.034
	FBI total score	-1.184	0.450	-0.621	0.386	6.922	1,11	0.023
	personal neglect	-14.127	3.020	-0.816	0.666	21.890	1,11	0.001
	impulsivity	-10.304	4.401	-0.577	0.333	5.842	1,11	0.039
**IADLs**	negative symptoms	-3.250	0.653	-0.832	0.693	24.785	1,11	<0.001
	FBI total score	-1.638	0.446	-0.742	0.551	13.506	1,11	0.004
	personal neglect	-14.443	4.191	-0.720	0.475	11.875	1,11	0.005
	hyperorality	-12.627	3.932	-0.696	0.484	10.316	1,11	0.008
**ADLs**	negative symptoms	-2.669	0.644	-0.781	0.610	17.188	1,11	0.002
	positive symptoms	-2.318	0.916	-0.607	0.368	6.408	1,11	0.028
	FBI total score	-1.443	0.387	-0.748	0.559	13.945	1,11	0.003
	personal neglect	-14.388	3.027	-0.820	0.673	22.593	1,11	0.001
	hyperorality	-10.091	3.699	-0.635	0.403	7.441	1,11	0.020
**hygiene**	negative symptoms	-3.437	1.066	-0.697	0.486	10.402	1,11	0.008
	positive symptoms	-4.227	1.066	-0.767	0.588	15.719	1,11	0.002
	FBI total score	-2.176	0.524	-0.781	0.611	17.249	1,11	0.002
	personal neglect	-20.455	4.493	-0.808	0.653	20.727	1,11	0.001
	impulsivity	-17.120	5.944	-0.656	0.430	8.295	1,11	0.015

**meal preparation**	aspontaneity	-20.588	7.904	-0.618	0.381	6.785	1,11	0.024
**telephoning**	personal neglect	-16.731	6.762	-0.598	0.358	6.122	1,11	0.031
	impulsivity	-16.869	7.064	-0.584	0.341	5.703	1,11	0.036
**outing**	negative symptoms	-3.609	0.591	-0.879	0.772	37.290	1,11	<0.001
	positive symptoms	-2.663	1.127	-0.580	0.337	5.581	1,11	0.038
	FBI total score	-1.832	0.429	-0.790	0.623	18.208	1,11	0.001
	disorganization	-14.395	4.547	-0.690	0.477	10.023	1,11	0.009
	hyperorality	-11.532	4.587	-0.604	0.365	6.320	1,11	0.029
**finance and correspondence**	negative symptoms	-4.857	0.912	-0.849	0.720	28.333	1,11	<0.001
	positive symptoms	-4.092	1.482	-0.640	0.409	7.622	1,11	0.019
	FBI total score	-2.595	0.581	-0.803	0.644	19.929	1,11	0.001
	disorganization	-23.642	5.087	-0.814	0.663	21.598	1,11	0.001
**medication**	negative symptoms	-3.802	1.325	-0.654	0.428	8.230	1,11	0.015
	personal neglect	-18.182	7.132	-0.609	0.371	6.498	1,11	0.027
**housework and leisure activities**	negative symptoms	-2.293	0.683	-0.712	0.506	11.280	1,11	0.006
	apathy	-19.524	3.504	-0.859	0.738	31.054	1,11	<0.001
	loss of insight	-9.457	4.025	-0.578	0.334	5.520	1,11	0.039

SE: Standard Error, df: degrees of freedom

Tables 9: Summary of the second set of stepwise regression analyses regarding the behavioral symptoms with the greatest contribution to ADL performance, as assessed by the FBI and DAD ratings.

**Dependent Variable**	**Steps**	**Predictive Factor**	**B**	**SE B**	**Standardized *β***	R^2	***F***	**df**	***p***
**ADLs**	1	personal neglect	-14.388	3.027	-0.820	0.673	22.593	1,11	0.001
	2	personal neglect	-9.650	3.054	-0.550	0.805	20.608	2,10	<0.001
		negative symptoms	-1.548	0.595	-0.453				

df: degrees of freedom; SE: Standard Error

Table 10: Summary of the second set of stepwise regression analyses regarding the behavioral symptoms with the greatest contribution to each functional domain, as assessed by the FBI and DAD ratings.

Dependent Variable	Predictive Factor	B	SE B	Standardized *β*	R^2	*F*	df	*p*
**BADLs**	personal neglect	-14.127	3.020	-0.816	0.666	21.890	1,11	0.001
**IADLs**	negative symptoms	-3.250	0.653	-0.832	0.693	24.785	1,11	<0.001
**hygiene**	personal neglect	-20.455	4.493	-0.808	0.653	20.727	1,11	0.001
**telephoning**	personal neglect	-16.731	6.762	-0.598	0.358	6.122	1,11	0.031
**outing**	negative symptoms	-3.609	0.591	-0.879	0.772	37.290	1,11	<0.001
**finance and correspondence**	negative symptoms	-4.857	0.912	-0.849	0.720	28.333	1,11	<0.001
**medication**	negative symptoms	-3.802	1.325	-0.654	0.428	8.230	1,11	0.015
**meal preparation**	aspontaneity	-20.588	7.904	-0.618	0.381	6.785	1,11	0.024

SE: Standard Error, df: degrees of freedom

Table 11: Summary of the second set of stepwise regression analyses regarding the behavioral symptoms with the greatest contribution to housework and leisure activities, as assessed by the FBI and DAD ratings.

**Dependent Variable**	**Steps**	**Predictive Factor**	**B**	**SE B**	**Standardized β**	R^2	F	**df**	p
**housework and leisure activities**	1	apathy	-19.524	3.504	-0.859	0.738	31.054	1, 11	<0.001
	2	apathy,	-17.011	3.197	-0.316	0.826	23.764	2, 10	<0.001
		loss of insight	-5.173	2.302					

SE: Standard Error, df: degrees of freedom

Table 12: Summary of the first set of stepwise regression analyses regarding the contribution of neuropsychological tests to compensatory strategy use, as assessed by the IADL-C ratings.

Dependent Variable	Predictive Factor	B	SE B	Standardized *β*	R^2	*F*	df	*p*
**Use of compensatory strategies**	FES total score	1,117	0.485	0.570	0.325	5.303	1,11	0.042
	semantic verbal fluency	0.906	0.264	0.719	0.517	11.784	1,11	0.006
	backward digit span	2.221	0.608	0.740	0.548	13.322	1,11	0.004
	TMT-Part B	-0.024	0.009	-0.615	0.379	6.706	1,11	0.025
	Stroop-words	0.132	0.049	0.629	0.395	7.197	1,11	0.021
	FL incongruent-correct	0.266	0.089	0.669	0.448	8.928	1,11	0.012
	Taylor-copy	0.265	0.113	0.578	0.334	5.526	1,11	0.038
	Taylor-delayed recall	0.741	0.239	0.683	0.467	9.642	1,11	0.010
	list learning-immediate recall	0.485	0.115	0.785	0.617	17.717	1,11	0.001
	confrontation naming test	0.266	0.117	0.565	0.320	5.166	1,11	0.044

FL incongruent-correct: the total number of incongruent trials in the Flanker test where the subject's response was correct (incongruent are the trials where the non-target arrows point in the opposite direction than the centrally presented target arrow),

SE: Standard Error, df: degrees of freedom

Table 13: Summary of the second set of stepwise regression analyses regarding the neuropsychological tests with the greatest contribution to compensatory strategy use, as assessed by the IADL-C ratings.

**Dependent Variable**	**Steps**	**Predictive Factor**	**B**	**SE B**	**Standardized *β***	R^2	***F***	**df**	***p***
**compensatory strategy use**	1	list learning-immediate recall	0.485	0.115	0.785	0.617	17.717	1,11	0.001
	2	list leaning-immediate recall,	0.352	0.095	0.570	0.809	21.139	2.10	<0.001
		backward digit span	4.465	0.463	0.488				

<u>SE</u>: Standard Error, <u>df</u>: degrees of freedom
